# In Vitro Characterization of an Equinized Anti-PD-L1 Antibody for Cancer Immunotherapy in Horses

**DOI:** 10.3390/vetsci13040343

**Published:** 2026-04-01

**Authors:** Takeru Horikawa, Naoya Maekawa, Tomohiro Okagawa, Wisa Tiyamanee, Otgontuya Ganbaatar, Hayato Nakamura, Mari Ikehata, Maho Inoue, Takeshi Nakanishi, Taro Tachibana, Yukinari Kato, Keiichi Yamamoto, Yasuhiko Suzuki, Shiro Murata, Kazuhiko Ohashi, Satoru Konnai

**Affiliations:** 1 Department of Disease Control, Faculty of Veterinary Medicine, Hokkaido University, Sapporo 060-0818, Japan; 2 Cancer Research Unit, One Health Research Center, Hokkaido University, Sapporo 060-0818, Japan; 3 Institute for Vaccine Research and Development (HU-IVReD), Hokkaido University, Sapporo 001-0021, Japan; 4 Business Development Unit, FASMAC Co., Ltd., Atsugi 243-0021, Japan; 5 Department of Chemistry and Bioengineering, Division of Science and Engineering for Materials, Chemistry and Biology, Graduate School of Engineering, Osaka Metropolitan University, Osaka 558-8585, Japan; 6 Department of Antibody Drug Development, Tohoku University Graduate School of Medicine, Sendai 980-8575, Japan; 7 Division of Bioresources, International Institute for Zoonosis Control, Hokkaido University, Sapporo 001-0020, Japan; 8 Global Station for Zoonosis Control, Global Institution for Collaborative Research and Education (GI-CoRE), Hokkaido University, Sapporo 060-0808, Japan; 9 Veterinary Research Unit, International Institute for Zoonosis Control, Hokkaido University, Sapporo 001-0021, Japan; 10 International Affairs Office, Faculty of Veterinary Medicine, Hokkaido University, Sapporo 060-0818, Japan

**Keywords:** equine cancer, immunotherapy, immune checkpoint inhibitor, programmed death ligand 1 (PD-L1), antibody drug

## Abstract

Horses develop spontaneous tumors, typically in old age. Although local tumor control can be achieved using conventional therapies, systemic therapies are required to treat recurrent and/or metastatic tumors. Immune checkpoint inhibitors, such as anti-PD-L1 antibodies, have been approved for the treatment of various tumor types in humans; however, little is known about the immunosuppressive roles of the PD-1/PD-L1 pathway in horses, and the therapeutic potential of these inhibitors remains to be elucidated. Previously, we reported that the rat monoclonal anti-PD-L1 antibody 6C11-3A11 cross-reacts with horse PD-L1 to block the PD-1/PD-L1 interaction. To further develop antibodies for therapeutic purposes, their immunogenicity must be reduced to maximize efficacy and safety. To this end, we designed an equinized (equine-ized) anti-PD-L1 antibody, Eq6C11, using the complementarity-determining regions of 6C11-3A11. Eq6C11 had antigen-binding properties comparable to those of 6C11-3A11 and inhibited equine PD-L1 binding to PD-1 in a recombinant protein-based assay. Treatment with Eq6C11 significantly increased IFN-γ and IL-2 production in equine peripheral blood mononuclear cell cultures, suggesting its stimulatory activity on T-cell activation. Although further studies are needed to clarify its immunogenicity and clinical activity, these results encourage further development of Eq6C11 as a candidate immune checkpoint inhibitor for cancer immunotherapy in horses.

## Simple Summary

Companion animals, including horses, develop spontaneous tumors that pose a significant threat to their quality of life. In human medicine, immunotherapy has emerged as an effective systemic treatment for cancers, and immune checkpoint inhibitors such as anti-PD-L1 antibodies have shown promise for various tumor types. However, no attempts have been made to develop immune checkpoint-inhibiting antibodies for cancer immunotherapy in horses. In this study, based on a previously reported monoclonal antibody that cross-reacts with equine PD-L1, an equinized anti-PD-L1 antibody was prepared and tested for its biological function in vitro. The equinized antibody bound sufficiently to recombinant equine PD-L1, blocked the binding of equine PD-L1 to PD-1, and most importantly enhanced cytokine production when added to equine peripheral blood mononuclear cell cultures. Taken together, the equinized anti-PD-L1 antibody presented here could be a promising candidate as an immune checkpoint inhibitor for cancer immunotherapy in horses.

## 1. Introduction

Companion animals, including horses, develop spontaneous tumors, typically in old age. For example, the most frequently reported tumor types include sarcoids, malignant melanoma, and squamous cell carcinoma [1,2,3]. Horse tumors are primarily treated by surgical resection; additionally, various treatment options such as cryotherapy, laser surgery, and local chemotherapy are available [4,5,6]. Despite successful local treatments, limited systemic treatments have been developed for recurrent and/or metastasized equine cancers, leaving room for improvements in cancer therapies to provide better veterinary care.

Immunotherapy is a promising treatment option for human cancers. Among them, immune checkpoint inhibitors, such as anti-programmed death-1 (PD-1) and anti-programmed death ligand 1 (PD-L1) antibodies, have been widely used to treat various human tumor types [7,8]. The coinhibitory receptor PD-1 is expressed on activated T cells and transmits inhibitory signals when bound to its ligands, PD-L1 and PD-L2. PD-L1 expression can be induced in various cell types, including hematopoietic and nonhematopoietic cells; importantly, its overexpression is commonly observed in tumor cells [9,10]. In the tumor microenvironment (TME), tumor antigen-specific T-cell responses are suppressed by the PD-1/PD-L1 pathway, allowing tumors to evade host immune surveillance. Indeed, PD-L1 expression is associated with shorter survival in patients with renal cell carcinoma and gastric cancer [11,12], suggesting that it plays a critical role in suppressing antitumor immunity. In contrast, as the interaction between PD-1 and PD-L1 is reversible, the PD-1/PD-L1 axis has been proposed as a druggable target for cancer immunotherapy. Antibody drugs that interrupt PD-1/PD-L1 binding have been developed, and their antitumor efficacy has been demonstrated in numerous clinical studies involving patients with various tumor types, including malignant melanoma, lung cancer, and renal cancer [13]. In horses, however, only a few studies on the PD-1/PD-L1 pathway have been reported, and further research is warranted to evaluate the therapeutic potential of antibody drugs that interfere with immune checkpoint receptor–ligand interactions.

Previously, we identified equine *PD-1* and *PD-L1* mRNA sequences and found considerable similarities in their deduced amino acid sequences among orthologs from other mammalian species, including humans. Recombinant equine PD-1 binds to equine PD-L1, supporting the functional conservation of these molecules in horses [14]. Immunohistochemical studies have revealed PD-L1 expression in equine malignant melanoma [14,15], sarcoids [16], and squamous cell carcinoma [17,18]. Although its immunosuppressive role in the TME has been suggested, the utility of PD-L1 expression as a prognostic biomarker of survival and the therapeutic efficacy of blocking antibodies in equine cancers remain to be elucidated.

Our previous study also revealed that a rat monoclonal antibody (clone 6C11-3A11), which was established by immunizing rats with bovine PD-L1, cross-reacts with equine PD-L1. 6C11-3A11 efficiently blocked equine PD-1/PD-L1 binding in a recombinant protein-based assay, and enhanced cytokine production in equine peripheral blood mononuclear cell (PBMC) cultures stimulated with a superantigen [14]. To further develop monoclonal antibodies for therapeutic purposes, the immunogenicity of rodent antibodies must be reduced to maximize their efficacy and safety. In human medicine, chimeric antibodies, which consist of variable and constant regions of rodent and human antibodies, respectively, and humanized antibodies, in which complementarity-determining regions (CDRs) of rodent antibodies are grafted onto the human antibody framework, have been developed and approved for cancer treatment [19]. By reducing the proportions of xenogeneic amino acid sequences, these engineered antibodies are less immunogenic in humans, allowing repeated administration with a decreased potential to induce anti-drug antibody (ADA) production. ADA may neutralize therapeutic activity, affect pharmacokinetics, and induce allergic reactions; thus, ADA induction thereby negatively influences the efficacy and safety of antibody drugs.

To this end, in this study, we designed an equinized (equine-ized) anti-PD-L1 monoclonal antibody using the CDRs of 6C11-3A11 and tested its binding to equine PD-L1 in comparison with 6C11-3A11 and the equine chimeric version of the monoclonal antibody. The therapeutic potential was evaluated using an inhibition assay against equine PD-1/PD-L1 binding and a PBMC cultivation assay as an indicator of T-cell activation.

## 2. Materials and Methods

### 2.1. Equine Blood Samples

This study was approved by the Institutional Animal Care and Use Committee of the Faculty of Veterinary Medicine, Hokkaido University (approval #20-0093 and #25-0070), which is fully accredited by the Association for Assessment and Accreditation of Laboratory Animal Care International. Peripheral blood samples were obtained from 31 healthy horses, including thoroughbreds (*n* = 26), a pony, and crossbreeds (*n* = 4), kept on private farms in Hokkaido, Japan. Characteristics of donor horses were summarized in Supplementary Table S1.

### 2.2. Cell Culture

ExpiCHO-S cells (Thermo Fisher Scientific, Waltham, MA, USA) were cultured in ExpiCHO Expression Medium (Thermo Fisher Scientific) at 37 °C and 8% CO_2_. Expi293F cells (Thermo Fisher Scientific) were cultured in Expi293 Expression Medium (Thermo Fisher Scientific) at 37 °C and 8% CO_2_. Equine PBMCs were obtained from heparinized blood by density gradient centrifugation on Percoll (Cytiva, Tokyo, Japan), and cultured in RPMI 1640 medium (Sigma-Aldrich, St. Louis, MO, USA) supplemented with 10% fetal bovine serum, 2 mM L-glutamine, 100 µg/mL streptomycin, and 100 U/mL penicillin (Thermo Fisher Scientific) at 37 °C and 5% CO_2_. A superantigen, Staphylococcal enterotoxin B from *Staphylococcus aureus* (SEB, Sigma-Aldrich), was added to the medium at a concentration of 0.1 µg/mL to mimic antigen stimulation of T cells.

### 2.3. Equinized or Equine Chimeric Anti-PD-L1 Monoclonal Antibody

The heavy and light chain variable regions of the equinized monoclonal antibody (Eq6C11) were designed using the CDR grafting method with the CDRs of the rat anti-bovine PD-L1 monoclonal antibody (clone: 6C11-3A11) [14,20]. CDRs of 6C11-3A11 light chain and heavy chain were identified and the amino acid sequences were grafted onto equine antibody frameworks to generate equinized variable regions of light and heavy chain [19,21]. The variable regions of Eq6C11 were combined with the equine kappa chain constant region (GenBank accession number X75612) and the equine IgG1 constant region (GenBank accession number AJ302055). To reduce antibody effector functions, amino acid mutations were introduced into Fcγ receptor- and complement-binding sites within the heavy chain constant region [22,23,24]. Similarly, an equine chimeric monoclonal antibody (Eqch6C11) was designed by combining the rat variable regions and the corresponding equine constant regions. The nucleotide sequences of the Eq6C11 and Eqch6C11 heavy and light chains were codon-optimized for expression in CHO cells, synthesized (GenScript Japan, Tokyo, Japan; Eurofins Genomics, Tokyo, Japan), and inserted into the expression vectors pCAGEN [25] or pDC62c5-U533 [26]. The plasmids were introduced into ExpiCHO-S cells using an ExpiFectamine CHO Transfection Kit (Thermo Fisher Scientific). The culture supernatant was harvested on day 10. Antibodies were purified from the culture supernatant by affinity chromatography using Ab-Capcher Extra (Protenova, Kagawa, Japan), and the buffer was exchanged with phosphate-buffered saline (PBS) using PD-MidiTrap G-25 (Cytiva). The antibody concentration was measured using a NanoDrop 8000 spectrophotometer (Thermo Fisher Scientific). Sodium dodecyl sulfate-polyacrylamide gel electrophoresis (SDS-PAGE) was performed under reducing and nonreducing conditions using a 5–20% gradient polyacrylamide gel (Wako, Osaka, Japan) and 2× Laemmli Sample Buffer (Bio-Rad, Hercules, CA, USA). Precision Plus Protein Standards Dual Color (Bio-Rad) was used as a molecular weight marker, and proteins were visualized with Coomassie Brilliant Blue (CBB) staining kit (Integrale, Tokushima, Japan).

### 2.4. Surface Plasmon Resonance (SPR) Analysis

The binding avidity of the prepared anti-PD-L1 antibodies to the polyhistidin-fused equine PD-L1 protein (EqPD-L1-His) was assessed in SPR analyses. For establishment of EqPD-L1-His, the cDNA of the putative ectodomain of equine PD-L1 was fused with 6 × polyhistidine and amplified by PCR [14,27], using gene-specific primers for C-terminal his tagging (5′-TTT GCT AGC CGC CAC CAT GAG GAT AGT TAG TGT CTT-3′ and 5′-CCC CTC GAG TTA ATG GTG ATG GTG ATG GTG GTG AGT TCT CTT ATT TGC CG-3′). The amplicon was purified and subcloned into the multiple cloning site of the pCXN2.1(+) vector (kindly provided by Dr. T. Yokomizo, Juntendo University, Japan) [28] and the resulting expression vector was transfected into Expi293F cells using an ExpiFectamine 293 Transfection Kit (Thermo Fisher Scientific). After cell culture for 7 days, EqPD-L1-His proteins were purified from the harvested supernatant using TALON Metal Affinity Resin (Clontech, Palo Alto, CA, USA), followed by the buffer exchange with PBS by ultrafiltration using an Amicon Ultra-4 Ultracel-3 (Merck Millipore, Billerica, MA, USA).

SPR analysis was carried out using a Biacore X100 system with a CM5 sensor chip and a His Capture Kit (Cytiva). After the immobilization of the anti-histidine antibody onto the sensor chip via amine coupling, EqPD-L1-His was captured on the chip, followed by the characterization of the binding kinetics of anti-PD-L1 antibodies. HBS-EP+ buffer (Cytiva) was utilized for both sample dilution and running buffer. To obtain the specific binding responses, the reference responses of the buffer-only control runs were subtracted. The kinetic constants of each antibody were calculated by fitting with the 1:1 kinetic binding model. At least three independent experiments were conducted and the data are shown as mean ± SE.

### 2.5. Flow Cytometry

To examine the ability of anti-PD-L1 antibodies to bind equine PD-L1 on the cell surface, flow cytometry was performed using recombinant equine PD-L1 expressing cells. To obtain cells that expressed equine PD-L1 on their surface, an expression vector encoding equine PD-L1 fused with enhanced green fluorescent protein (EGFP) [14] was introduced into ExpiCHO-S cells and cultured overnight. Subsequently, 2 × 10^5^ cells were incubated with Eqch6C11 or Eq6C11 at the indicated concentrations (100 pg to 10 µg/mL) at room temperature for 30 min. The cells were washed twice and then incubated with Alexa Fluor 647-conjugated goat polyclonal anti-horse IgG antibody (Jackson ImmunoResearch, West Grove, PA, USA) at room temperature for 20 min. Cells were washed twice and analyzed using a FACS Lyric flow cytometer (BD Biosciences, San Jose, CA, USA). After the cell population was identified by gating based on the side and forward scatter, antibody binding was detected using Alexa Fluor 647 fluorescence within the EGFP-positive cell population. To ensure reproducibility, three independent experiments were conducted and representative data are shown in the histograms.

### 2.6. Binding Inhibition Assay of Equine PD-1/PD-L1

To evaluate the PD-1/PD-L1 blockade by the anti-PD-L1 antibodies, a binding inhibition assay was performed in a 96-well plate with the rabbit IgG Fc-tagged equine PD-1 and PD-L1 proteins (EqPD-1-Ig and EqPD-L1-Ig) [14]. EqPD-L1-Ig was biotinylated using a Biotin Conjugation Kit (Fast, Type A)-Lightning Link (Abcam, Cambridge, UK). EqPD-1-Ig was coated on a Maxisorp flat-bottomed microwell plate (Thermo Fisher Scientific) and the plate was blocked with SuperBlock T20 (PBS) blocking buffer (Thermo Fisher Scientific). Biotinylated EqPD-L1-Ig (0.1 µg/mL) was preincubated with Eqch6C11 or Eq6C11 at various concentrations (0.1, 1, 2.5, 5, 7.5, or 10 μg/mL) for 30 min at 37 °C and added to the coated plate. The binding of equine PD-L1 to PD-1 was visualized using NeutrAvidin-horseradish peroxidase (Thermo Fisher Scientific) and TMB One-Component Substrate (Bethyl Laboratories, Montgomery, TX, USA). The reaction was stopped by adding 0.18 M H_2_SO_4_, and the optical density (OD) at 450 nm was measured using a microplate reader MTP-900 (Corona Electric, Ibaraki, Japan). Relative OD (%OD) was calculated from the OD of the tested sample in comparison with that of the control reaction developed without the addition of blocking antibody (0 μg/mL). For the negative control, Equine IgG (Jackson ImmunoResearch) was substituted for the primary antibody. The experiment was independently repeated three times and the data are shown as mean ± SD. Statistical analysis was performed by one-way analysis of variance (ANOVA) followed by Tukey’s honest significant difference (HSD) test.

### 2.7. Functional Blockade of Equine PD-L1 in PBMC Cultures

To assess the functional enhancement of equine immune cells by the blockade of the PD-1/PD-L1 interaction, equine PBMCs were cultured in the presence of 0.1 μg/mL SEB and treated with 10 µg/mL of anti-PD-L1 antibodies. Equine IgG (Jackson ImmunoResearch) was used as the negative control. PBMCs culture was conducted in triplicate for each experimental condition. To evaluate cytokine production from cultured PBMCs, the culture supernatant was harvested on day 3, and the concentrations of interferon gamma (IFN-γ) and interleukin 2 (IL-2) were measured by enzyme-linked immunosorbent assay (ELISA) using the Equine IFN-γ ELISA Development Kit (HRP) (Mabtech, Nacka Strand, Sweden) and the Equine IL-2 DuoSet ELISA (R&D Systems, Minneapolis, MN, USA), respectively. To evaluate the effect of our antibody on the CD4^+^ or CD8^+^ lymphocytes proliferation, we measured the incorporation of nucleotide analogue using Click-iT Plus EdU Alexa Fluor 647 Flow Cytometry Assay Kit (Thermo Fisher Scientific). PBMC cultures for the cell proliferation assay were conducted in duplicate for each condition. The thymidine analogue, 5-Ethynyl-2′-deoxyuridine (EdU), was added to the culture medium at a final concentration of 10 μM on day 3, followed by the incubation for an additional 2 h. The cells harvested after incubation were labeled with optimal concentrations of anti-equine CD4 antibody conjugated with FITC (Bio-Rad) and anti-equine CD8 antibody conjugated with PE (Bio-Rad) at room temperature for 30 min. Subsequently, incorporated EdU was fluorescently labeled with Alexa Fluor 647 following the instructions of the kit. Fluorescence of the cells was analyzed with a FACS Lyric flow cytometer (BD Biosciences). EdU incorporation in CD4^+^ cells or CD8^+^ cells was evaluated within the lymphocyte population gated using forward scatter and side scatter. Statistical analyses were performed using the Wilcoxon signed-rank test. *p* values were adjusted for multiple testing using Holm’s adjustment method.

### 2.8. Statistical Analyses

All statistical tests were performed using R version 4.3.2 [29]. A *p* value of less than 0.05 was considered statistically significant.

## 3. Results

### 3.1. Preparation of Equinized Anti-PD-L1 Monoclonal Antibody (Eq6C11)

**Figure 1:**
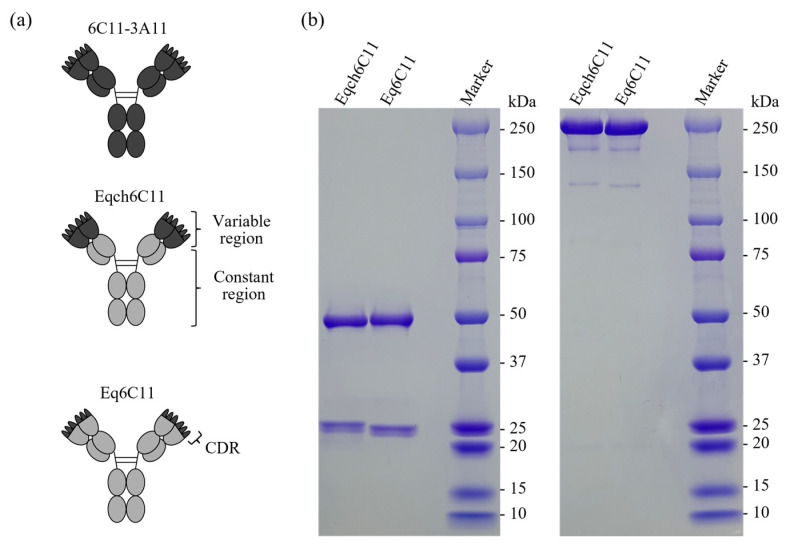
Preparation of equinized or equine chimeric anti-PD-L1 antibody. (**a**) Schematic image of the rat monoclonal (clone 6C11-3A11), equine chimeric (Eqch6C11), and equinized (Eq6C11) antibodies. Dark grey regions represent rat protein sequences, and light grey regions represent horse protein sequences. (**b**) SDS-PAGE analysis of the produced antibodies. Proteins were visualized using CBB staining. Left panel, reducing condition; right panel, nonreducing condition.

The CDRs form hypervariable loops that interact with specific antigens. Thus, CDR grafting onto a human antibody framework has been widely used to prepare humanized antibodies. We applied this technique to prepare an equinized antibody (Eq6C11) using the CDRs of the cross-reactive rat anti-PD-L1 monoclonal antibody 6C11-3A11. For comparison, the equine chimeric version of the same monoclonal antibody (Eqch6C11) was also prepared by combining the variable regions of 6C11-3A11 and the constant regions of the equine IgG1/kappa. The resulting antibodies had reduced proportions of xenogeneic amino acid sequences, with Eqch6C11 being 65% and Eq6C11 being 87% equine protein sequences based on the amino acid sequence identity (Figure 1a).

Eqch6C11 and Eq6C11 were produced as recombinant proteins using a mammalian cell-based transient expression system and purified from the culture supernatant using protein A affinity chromatography. SDS-PAGE under reducing conditions revealed the presence of a putative heavy chain (approximately 50 kDa) and a light chain (approximately 25 kDa) in both antibodies. Under nonreducing conditions, a single band was observed at 150–250 kDa, suggesting the formation of an antibody heterotetramer consisting of two heavy chains and two light chains linked together by interchain disulfide bonds (Figure 1b) as expected for intact IgG molecules. Although a few extra bands were visible under nonreducing conditions, the amount was comparable between the two antibodies and was negligible for further in vitro characterization of the antibodies (the original pictures can be found in Figure S1).

### 3.2. Eq6C11 Had Similar Binding Properties to Recombinant Equine PD-L1

To examine the binding properties of Eq6C11, SPR analysis was performed using recombinant equine PD-L1 extracellular domain (EqPD-L1-His) as the ligand. The association rate constant (k_a_), dissociation rate constant (k_d_) and equilibrium dissociation constant (K_D_) of Eq6C11 were 4.75 × 10^5^/Ms, 3.50 × 10^−4^/s, and 7.43 × 10^−10^ M, respectively, which were similar to those of 6C11-3A11 and Eqch6C11 (Table 1) indicating comparable binding kinetics. The sub-nanomolar K_D_ values suggest that these antibodies bind to equine PD-L1 with high affinity, even though 6C11-3A11 was originally raised against bovine PD-L1.

**Figure 2:**
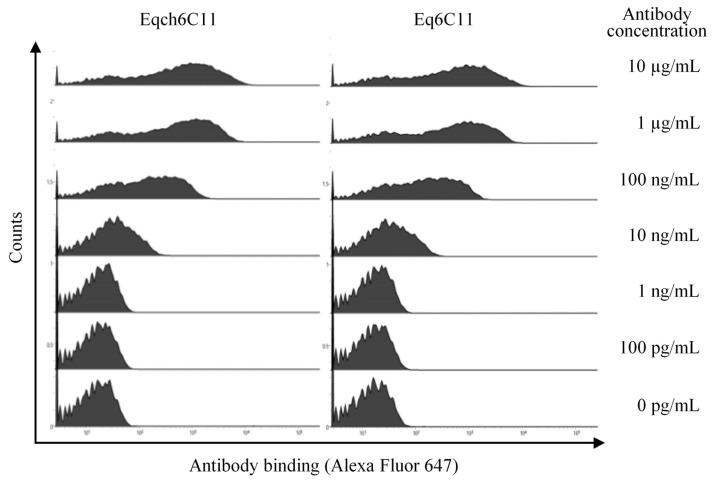
Binding of Eqch6C11 or Eq6C11 to cell surface equine PD-L1. Bindings of anti-PD-L1 antibodies (Eqch6C11, **left**; Eq6C11, **right**) to equine PD-L1-EGFP-expressing cells. ExpiCHO-S cells were transfected with the expression vector and the binding of the primary antibody was detected using Alexa Fluor 647-conjugated anti-horse IgG antibody within the EGFP-positive cell population.

Next, we tested whether Eq6C11 efficiently bound to equine PD-L1 expressed on the cell surface. To this end, equine PD-L1-expressing cells (tagged with EGFP at the C-terminus of the PD-L1 intracellular tail) [14] were prepared, and antibody binding was detected by flow cytometry after incubation with serially diluted antibodies. Both Eqch6C11 and Eq6C11 bound to equine PD-L1-expressing cells (EGFP^+^ cells) in a concentration-dependent manner and this binding was detectable at concentrations as low as 10 ng/mL (Figure 2). Taken together, we concluded that Eq6C11 has sufficient binding properties to equine PD-L1 and can be used for further functional assessment of its therapeutic potency.

### 3.3. Eq6C11 Blocked Equine PD-1/PD-L1 Binding and Enhanced Cytokine Production from Equine PBMCs

**Figure 3:**
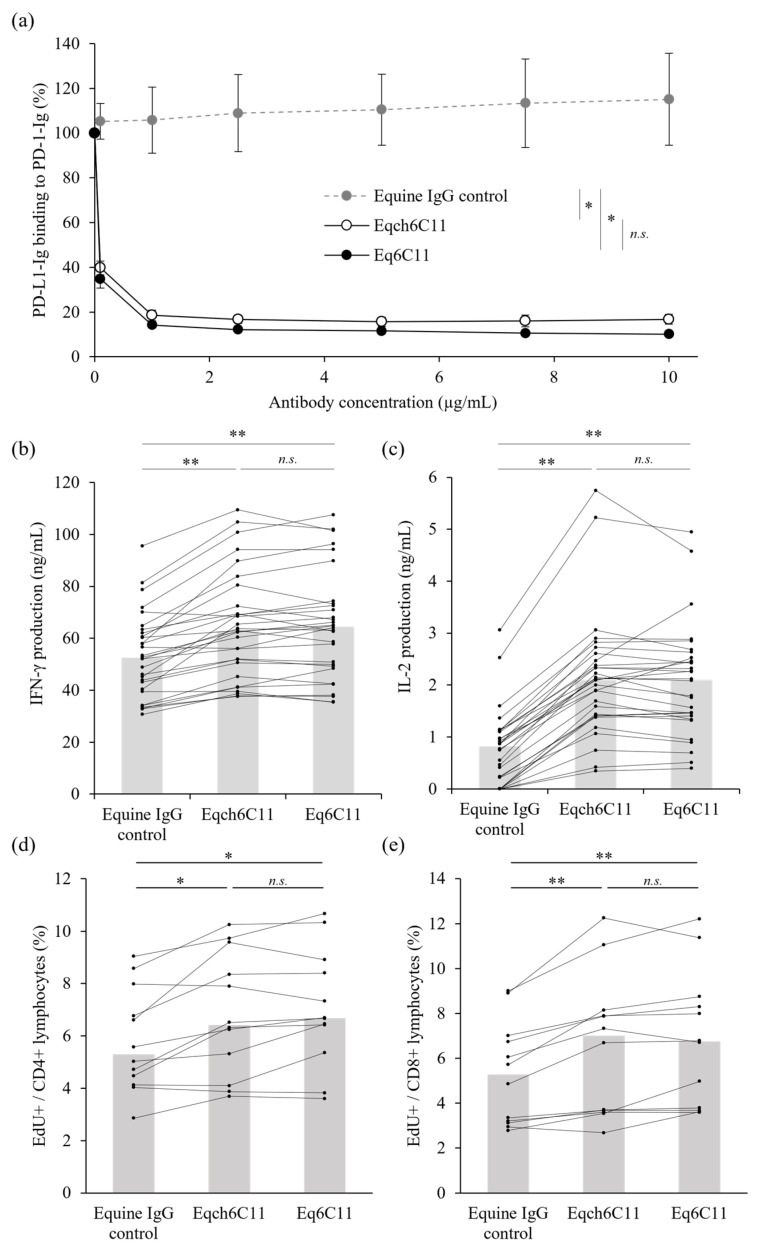
Binding inhibition of equine PD-1/PD-L1 and enhancement of cytokine production and cell proliferation by Eq6C11 treatment. (**a**) The blocking activity of anti-PD-L1 antibodies on the binding of EqPD-L1-Ig to plate-coated EqPD-1-Ig. Equine IgG was used as negative control antibody. The relative OD (%OD) was calculated in relation to the control incubated without blocking antibody. Each point indicates the average value of three independent experiments. Error bars indicate SD. Statistical analysis was performed using one-way ANOVA followed by Tukey’s HSD test for multiple comparison. An asterisk (*) indicates *p* < 0.01 at all antibody concentrations tested (0.1, 1, 2.5, 5, 7.5, and 10 μg/mL). *n.s.*, not significant. (**b**,**c**) Enhancement of cytokines production in equine PBMC cultures. Equine PBMCs (*n* = 30) were obtained from healthy horse donors and stimulated with 0.1 μg/mL SEB in the presence of 10 μg/mL anti-PD-L1 antibody (Eqch6C11 or Eq6C11). Equine IgG was used as a control antibody. Culture supernatants were harvested on day 3, and concentration of (**b**) IFN-γ or (**c**) IL-2 was measured by ELISA. (**d**,**e**) Enhancement of cell proliferation in equine PBMC cultures. Equine PBMCs (*n* = 12) collected from clinically healthy horses were cultured in the same condition as the cytokines production assay for 3 days. To evaluate cell proliferation, PBMCs were incubated with EdU during the last 2 h before the harvest. The lymphocyte population was gated by forward scatter and side scatter, and the incorporation of EdU in (**d**) CD4^+^ or (**e**) CD8^+^ cells was evaluated by a flow cytometer. (**b**–**e**) The gray bar represents the median value of each treatment group. Statistical analysis was performed using the Wilcoxon signed-rank test with Holm’s adjustment for multiple comparison. A single asterisk (*) or a double asterisk (**) indicate *p* < 0.05 or *p* < 0.01 respectively. *n.s.*, not significant.

To investigate whether Eq6C11 can be used for cancer immunotherapy in horses, the binding inhibition activity of equine PD-1/PD-L1 was evaluated using a recombinant protein-based assay. To this end, the extracellular domain of equine PD-1 (fused to rabbit IgG Fc) was coated on a microwell plate, and binding of the extracellular domain of equine PD-L1 (fused to rabbit IgG Fc, biotinylated) was detected on the plate using avidin-HRP and substrate reactions. As expected, the irrelevant equine IgG control (purified from the serum of non-immunized horses) did not affect equine PD-1/PD-L1 binding, whereas Eqch6C11 and Eq6C11 significantly reduced PD-L1 binding to PD-1 in a dose-dependent manner (Figure 3a). There was no significant difference in the inhibitory activities of Eqch6C11 and Eq6C11, which was consistent with their comparable binding kinetics.

Finally, to assess the immunostimulatory activity of Eq6C11, equine PBMCs obtained from healthy horses (*n* = 30) were cultured for three days with superantigen stimulation. Treatment with Eq6C11 significantly increased IFN-γ and IL-2 concentrations in the culture supernatant of stimulated equine PBMCs compared to the irrelevant equine IgG control. Notably, the increase in cytokine production was comparable to that observed after Eqch6C11 treatment (Figure 3b,c) consistent with their similar blocking activities. In addition, lymphocyte proliferation in a similar PBMC culture (*n* = 12) was evaluated by nucleotide analogue incorporation. Treatment with Eqch6C11 or Eq6C11 enhanced the proliferative capacity of both CD4^+^ and CD8^+^ lymphocytes (Figure 3d,e). Collectively, these results strongly suggest that Eq6C11 enhances T-cell activation by interrupting the PD-1/PD-L1 interaction, and that Eq6C11 is a promising candidate for equine cancer immunotherapy.

## 4. Discussion

In this study, we successfully produced an equinized anti-PD-L1 antibody using the CDR grafting method with unaltered binding properties to equine PD-L1, inhibitory activity against equine PD-1/PD-L1 binding, and immunostimulatory properties in horse immune cell cultures. The resulting equinized antibody contained approximately 90% horse protein sequences and possessed sufficient characteristics to pharmacologically block the PD-1/PD-L1 interaction. Thus, we propose that Eq6C11 could be a promising candidate as an immune checkpoint inhibitor for equine cancer immunotherapy. However, it should be noted that the immunostimulatory effects were examined only in healthy peripheral blood lymphocytes in this study, and the immunomodulatory effects of Eq6C11 within the TME remain unclear. Future investigations should therefore address TME-related aspects, for example by performing stimulation assays on tumor-infiltrating lymphocytes. In parallel, clinical studies will be necessary to further clarify the therapeutic efficacy of Eq6C11.

Eq6C11 and Eqch6C11 are designed based on equine IgG1 and κ chains. Equine IgG1 can functionally bind to Fcγ receptors (FcγRs) and C1q, like human IgG1. Additionally, its high affinity for protein A suggests a practical advantage in the development of therapeutic antibodies for horses [30]. Equine κ chain was selected over the lambda (λ) chain due to the use of κ chain in the original rat antibody 6C11-3A11. Furthermore, in alignment with human anti-PD-L1 antibodies (atezolizumab and durvalumab) which utilize human IgG1 and κ chains, equine IgG1 and κ chains were adopted for antibody production. In addition, the FcγR- and complement-binding sites of equine IgG1 are mutated for Eq6C11 and Eqch6C11 by changing conserved key residues known to interact with FcγRs and C1q [22,23,24]. The mutated IgG1 is expected to exert reduced antibody-dependent cellular cytotoxicity (ADCC) and complement-dependent cellular cytotoxicity (CDC), thereby sparing PD-L1-positive immune cells from antibody-mediated depletion. Because the effects of introduced mutations on ADCC or CDC were not evaluated in the current study, further investigation is warranted to confirm reduced effector functions of the mutated IgG1. Given that IgG1 is not the predominant subclass [30,31,32] and κ light chain is less abundant than λ light chain in equine serum [33], the possibility remains that other IgG subclasses, such as IgG3 or IgG4, or λ light chain may also be suitable for alternative therapeutic frameworks. Further studies are required to identify the most effective and safe antibody formats for equine medicine.

In human medicine, as antibody drugs that block the PD-1/PD-L1 axis, humanized anti-PD-1 antibody (pembrolizumab) and humanized anti-PD-L1 antibody (atezolizumab) have been widely used to treat various cancer types [34]. Eq6C11 is considered comparable to these approved antibody drugs in regard to the proportions of xenogeneic amino acid sequences; however, its immunogenicity must be evaluated in horses by carefully monitoring the emergence of ADA and the pharmacokinetics, especially when administered repeatedly. The frequency of allergic reactions may also serve as a surrogate marker of the immunogenicity of the test drug. In veterinary medicine, our group has demonstrated in veterinary clinical studies that the canine chimeric anti-PD-L1 antibody c4G12 exhibits antitumor efficacy against various cancer types in dogs and that the frequency of allergic reactions is reasonably low [27,35,36], supporting the further development of chimeric antibodies for canine cancer immunotherapy. In this regard, together with the fact that several chimeric antibodies are already used in human medicine, Eqch6C11 may serve as an alternative candidate for further development as an equine immune checkpoint inhibitor. Nonetheless, because Eq6C11 was comparable to Eqch6C11 in all aspects examined in this study, Eq6C11 is a preferred candidate with a theoretically lower immunogenicity.

PD-L1 expression has been demonstrated at high positive rates in equine malignant melanoma and squamous cell carcinoma [14,15,18], implying that these tumor types may benefit from anti-PD-L1 therapy. In human medicine, atezolizumab has been approved for *BRAF*^V600^ mutation-positive unresectable or metastatic melanoma in combination with cobimetinib and vemurafenib [13], and a recent phase 1 trial demonstrated the promising antitumor efficacy of cosibelimab, another anti-PD-L1 antibody, in patients with metastatic cutaneous squamous cell carcinoma [37], highlighting the clinical relevance of PD-L1–targeted therapies. Anti-PD-L1 therapy has been used to treat various cancer types in humans, such as non-small-cell lung cancer, urothelial carcinoma, and hepatocellular carcinoma [13,34]. Furthermore, clinical efficacy of anti-PD-L1 antibody has been demonstrated against several canine cancers, such as malignant melanoma and nasal adenocarcinoma, in previous veterinary clinical studies [27,35,36]. Therefore, other equine tumor types require further expression analysis of the target molecule. The rat monoclonal antibody 6C11-3A11 has been used for immunohistochemistry in equine tumor tissues with apparently high detection sensitivity [14]; thus, it could serve as a companion diagnostic tool for selecting animals eligible for anti-PD-L1 therapy. Further immunological analyses of the equine TME are urgently required to delineate the immunosuppressive mechanisms mediated by PD-1/PD-L1 in tumor antigen-specific T-cell responses. Unfortunately, no cross-reactive anti-PD-1 antibody is commercially available, and we were unable to analyze PD-1 expression in blood-circulating and tumor-infiltrating equine T cells. The development of such analytical tools will facilitate our understanding of antitumor immunity and immunotherapeutic opportunities in horses. Moreover, it has become promising options for cancer therapy to combine immune checkpoint inhibitors, including anti-PD-L1 antibodies, with radiotherapy and chemotherapy [38]. Combination with radiotherapy has been suggested to be effective for canine cancer treatment [35]. In future research, the evaluation of combination therapies is warranted for equine cancer therapy.

The immunostimulatory effects, antitumor efficacy, and safety profile of Eq6C11 should be evaluated in veterinary clinical studies involving horses with tumors. As the PD-1/PD-L1 axis serves as a negative regulator of T-cell activation and plays a pivotal role in maintaining T-cell tolerance to self [39], the blockade of this pathway may lead to autoimmune or immune-mediated toxicities. Indeed, cutaneous, gastrointestinal, renal, nervous, endocrine, musculoskeletal, hematological, and lung toxicities are commonly reported as immune-related adverse events (irAEs) associated with human immune checkpoint inhibitors [40], highlighting the need for careful monitoring in veterinary applications. In future studies, as the first step, a safety assessment will be conducted in a small group of horses to evaluate adverse events and determine the maximum tolerated dose. Once an acceptable dose has been established, clinical studies in horses with spontaneous tumors will be performed. Through these clinical investigations, we will evaluate antitumor responses and carefully monitor adverse events, including irAEs, to elucidate both the therapeutic efficacy and safety profile of Eq6C11.

In conclusion, we successfully prepared the equinized anti-PD-L1 antibody Eq6C11 for further development of cancer immunotherapy in horses. As a next step, veterinary clinical studies are needed to clarify its immunogenicity and evaluate its antitumor efficacy and safety in tumor-bearing horses. To the best of our knowledge, this is the first report on the development of an equinized antibody for cancer therapy in horses, which we believe will become an important step for improving veterinary care for animals suffering from neoplasms and advancing equine immuno-oncology.

## 5. Patents

N.M., T.O., Y.S., S.M., K.O., and S.K. are the authors of patent applications covering materials and techniques described in this paper (PCT/JP2018/011895 and PCT/JP2019/018899).

## Figures and Tables

**Table 1:** Binding properties of anti-PD-L1 antibodies to recombinant equine PD-L1.

Antibody	k_a_ (×10^5^/Ms)	k_d_ (×10^−4^/s)	K_D_ (×10^−10^ M)
6C11-3A11	5.95 ± 0.72	4.86 ± 0.19	8.66 ± 1.38
Eqch6C11	5.32 ± 0.74	2.91 ± 0.26	5.71 ± 1.08
Eq6C11	4.75 ± 0.13	3.50 ± 0.45	7.43 ± 1.12

Data are shown as mean ± SE of three or four independent experiments. k_a_, association rate constant; k_d_, dissociation rate constant; K_D_, equilibrium dissociation constant.

## Data Availability

The original contributions presented in this study are included in the article/Supplementary Materials. Further inquiries can be directed to the corresponding author.

## Supplementary Materials

The following are available online at https://www.mdpi.com/article/10.3390/vetsci13040343/s1, Figure S1: The original image of the SDS-PAGE gel., Table S1: Profiles of the horses for blood sampling.

## Abbreviations

The following abbreviations are used in this manuscript:

ADA	Anti-drug antibody
ANOVA	Analysis of variance
CBB	Coomassie Brilliant Blue
CDR	Complementarity-determining region
EdU	5-Ethynyl-2′-deoxyuridine
EGFP	Enhanced green fluorescent protein
ELISA	Enzyme-linked immunosorbent assay
HSD	Honest significant difference
IFN-γ	Interferon gamma
IL-2	Interleukin 2
irAE	Immune-related adverse event
OD	Optical density
PBMC	Peripheral blood mononuclear cell
PBS	Phosphate-buffered saline
PD-1	Programmed death 1
PD-L1	Programmed death ligand 1
SDS-PAGE	Sodium dodecyl sulfate–polyacrylamide gel electrophoresis
SEB	Staphylococcal enterotoxin B
SPR	Surface plasmon resonance
TME	Tumor microenvironment
