# Supplementary material for: In Vitro Characterization of an Equinized Anti-PD-L1 Antibody for Cancer Immunotherapy in Horses
Source: Vet Sci. 2026 Apr 1;13(4):343. doi: 10.3390/vetsci13040343 (PMC13119938; doi:10.3390/vetsci13040343)
Supplement: Supplementary file 1 [file vetsci-13-00343-s001.zip › vetsci-4116510-supplementary.pdf]

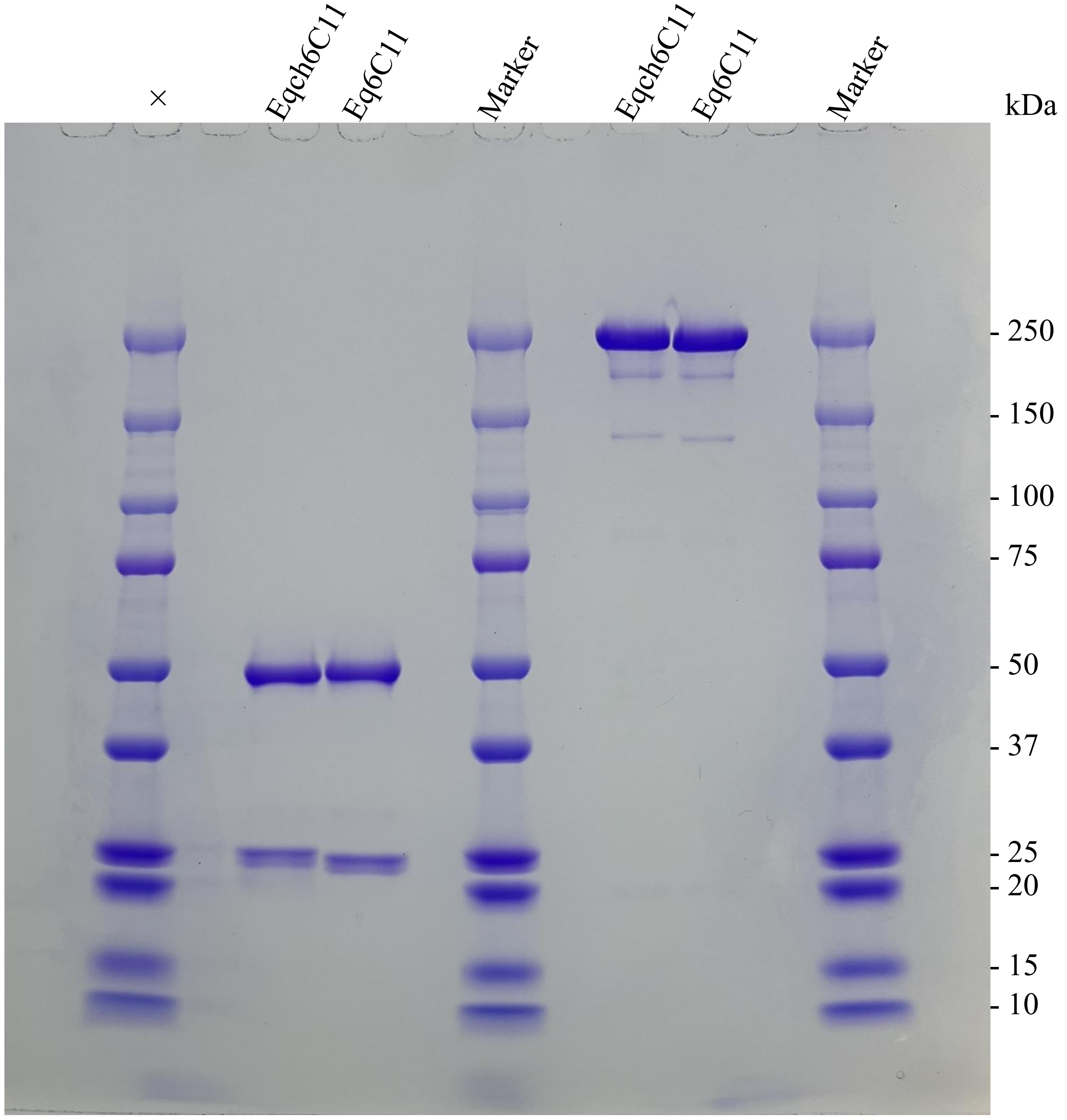

Figure S1. The original image of the SDS-PAGE gel.
The original uncropped and unadjusted image of the SDS-PAGE gel related to Fig 1b.

Table S1. Profiles of the horses for blood sampling

Sample	Sex	Age (year)	Breed	Experiments
#101	M	17	Thoroughbred	Cytokine, Proliferation
#102	M	14	Thoroughbred	Cytokine, Proliferation
#103	M	15	Thoroughbred	Cytokine, Proliferation
#104	M	20	Thoroughbred	Cytokine, Proliferation
#105	M	13	Thoroughbred	Cytokine, Proliferation
#106	M	16	Thoroughbred	Cytokine, Proliferation
#107	M	7	Thoroughbred	Cytokine, Proliferation
#108	M	12	Thoroughbred	Cytokine
#109	M	27	Thoroughbred	Cytokine
#110	M	10	Crossbreed	Cytokine, Proliferation
#111	F	12	Crossbreed	Cytokine, Proliferation
#112	F	4	Crossbreed	Cytokine, Proliferation
#113	M	20	Crossbreed	Proliferation
#114	M	18	Pony	Cytokine, Proliferation
#201	F	11	Thoroughbred	Cytokine
#202	F	16	Thoroughbred	Cytokine
#203	F	8	Thoroughbred	Cytokine
#204	F	9	Thoroughbred	Cytokine
#205	F	17	Thoroughbred	Cytokine
#206	F	10	Thoroughbred	Cytokine
#207	F	19	Thoroughbred	Cytokine
#301	F	13	Thoroughbred	Cytokine
#302	F	14	Thoroughbred	Cytokine
#303	F	12	Thoroughbred	Cytokine
#304	F	10	Thoroughbred	Cytokine
#305	F	10	Thoroughbred	Cytokine
#306	F	8	Thoroughbred	Cytokine
#307	F	9	Thoroughbred	Cytokine
#308	F	8	Thoroughbred	Cytokine
#309	F	10	Thoroughbred	Cytokine
#310	F	10	Thoroughbred	Cytokine

Peripheral blood samples were collected from 31 healthy horses. The age, sex, and breed of the horses are shown. M, male; F, female; Cytokine, cytokine production assay; Proliferation, cell proliferation assay.
